# 

**Published:** 1870-02

**Authors:** 


*The Thirtieth Annual Commencement of the Baltimore College of Dental Surgery*, will be held at the "Concordia Building," Baltimore, on Wednesday evening, March, 2nd, 1870. The Alumni and friends of the institution are cordially invited to be present.

